# Selective Detoxification of Phenols by *Pichia pastoris* and *Arabidopsis thaliana* Heterologously Expressing the PtUGT72B1 from *Populus trichocarpa*

**DOI:** 10.1371/journal.pone.0066878

**Published:** 2013-06-26

**Authors:** Zhi-Sheng Xu, Ya-Qiu Lin, Jing Xu, Bo Zhu, Wei Zhao, Ri-He Peng, Quan-Hong Yao

**Affiliations:** 1 College of Horticulture, Nanjing Agricultural University, Nanjing, Jiangsu, China; 2 Agricultural Biotechnology Research Center, Shanghai Academy of Agricultural Sciences, Shanghai, China; Lawrence Berkeley National Laboratory, United States of America

## Abstract

Phenols are present in the environment and commonly in contact with humans and animals because of their wide applications in many industries. In a previous study, we reported that uridine diphosphate-glucose-dependent glucosyltransferase PtUGT72B1 from *Populus trichocarpa* has high activity in detoxifying trichlorophenol by conjugating glucose. In this study, more experiments were performed to determine the substrate specificity of PtUGT72B1 towards phenolic compounds. Among seven phenols tested, three were glucosylated by PtUGT72B1 including phenol, hydroquinone, and catechol. Transgenic *Arabidopsis* plants expressing the enzyme PtUGT72B1 showed higher resistance to hydroquinone and catechol but more sensitivity to phenol than wild type plants. Transgenic *Pichia pastoris* expressing PtUGT72B1 showed enhanced resistance to all three phenols. Compared with wild type *Arabidopsis* plants, transgenic *Arabidopsis* plants showed higher removal efficiencies and exported more glucosides of phenol, phenyl β-D-glucopyranoside, to the medium after cultured with the three phenols. Protein extracts from transgenic *Arabidopsis* plants showed enhanced conjugating activity towards phenol, hydroquinone and catechol. PtUGT72B1 showed much higher expression level in *Pichia pastoris* than in *Arabidopsis* plants. Kinetic analysis of the PtUGT72B1 was also performed.

## Introduction

Phenols are widely distributed in the environment because of their various applications in several industrial processes. They can induce genotoxic [1], carcinogenic [2], and immunotoxic [3] effect. Phenol has been included in the lists of 11 priority pollutants by the US Environmental Protection Agency and the European Union. Hydroquinone can occur in several plant species or be artificially synthesized for wide commercial applications. Hydroquinone induces rats to produce renal tubule adenomas and to exacerbate spontaneous chronic progressive nephropathy [4]. Catechol can cause eczematous dermatitis in humans, depression of the central nervous system, and prolonged rise in blood pressure in animals. Many reactions can occur between catechol and biomolecules (DNA and proteins) or membranes, causing the latter to break, inactivate, or destruct [5].

Glycosyltransferases (GTs; EC 2.4.x.y) found in organisms across all Phyla are now divided into 94 distinct families (http://www.cazy.org/GlycosylTransferases.html). Uridine diphosphate (UDP)-glycosyltransferases (UGTs) belong to family 1 GTs and can transfer UDP-activated sugar to low molecular weight substrates. Over 120 putative UGT genes have been identified in *Arabidopsis thaliana* [6], [7]. While UDP-glucose (UDPG) is the most common activated sugar form observed in plants, UDP-rhamnose and UDP-xylose have also been determined [7]. Plant UGTs can glycosylate diverse endogenous substrates such as (iso)flavonoids [8]–[13], terpenes [14], auxin [15]–[17], salicylic acid [18], brassinosteroids [19], and sterols [20]. In addition, they possess a side-activity towards phytotoxic and xenobiotic, such as 2,4,6-trinitrotoluene [21], 2,4,5-trichlorophenol [22]–[24], 2,4-dichloroaniline [24]–[26], deoxynivalenol [23], [27] and zearalenone [28]. The glycosylation of the acceptors can occur at -OH, -COOH, -NH_2_, [25] -SH, and C-C groups [29].

The xenobiotic glycosylation ability of UGTs is also known to function in phase II detoxification pathways in plants. The activity of PtUGT72B1 in detoxifying trichlorophenol (TCP) in *Arabidopsis* by conjugating glucose has been previously identified [22]. In this paper, we aim to analyze the substrate specificity of UGT72B1 towards phenols and to determine whether the UGT72B1 can enhance the phytodegradation ability of *Arabisopsis* plants and *P. Pastoris* to phenolic pollutants.

## Materials and Methods

### *PtUGT72B1* Expression in *Pichia pastoris* and Activity Assays

*PtUGT72B1* gene (GenBank Accession No: XP_002320190) was transformed into *P. pastoris* strain GS115 cells by electroporation [22]. Briefly, the transformants selected from histidine-deficient SD medium after incubated at 30°C for 3 d were cultured in BMGY medium for 24 h, and then induced in BMMY medium supplemented with 1% methanol at 30°C and constant shaking at 200 rpm for 24 h. To extract the expressed PtUTG72B1 protein from *P. pastoris*, 100 ml of 1-day induced yeast was centrifuged and washed twice with sterilized distilled water. After harvested by centrifugation, the cells were flash-frozen in liquid nitrogen for subsequent grinding to a fine powder in the presence of ice, and resuspended in 4 mL of buffer consisting of 50 mM phosphate-buffered saline (PBS, pH 7.4), 20% glycerol, 1 mM 1,4-dithiothreitol (DTT), 0.1 mM ethylene diamine tetraacetic acid (EDTA). Cell debris was removed by centrifugation at 20,000 g for 20 min at 4°C. The supernatant was used as crude enzyme extract and stored at −20°C. Protein from *P. pastoris* containing pPIC9K empty vector was also extracted and used as a control.

Seven phenols were selected, including phenol, hydroquinone, catechol, 3-methylcatechol, 8-hydroxyquinoline, 4-nitrophenol, and bisphenol A. To determine the glucosides of phenols resulting from enzymatic transformation by PtUGT72B1, GT activity was assayed in a mixture containing 10 µg of enzyme extract, 50 mM PBS (pH 7.4), 2 mM UDP-glucose, and 0.2 mM acceptor substrate (dissolved in ethanol). The reactions were carried out in 200 µL volumes at 30°C for 1 h, stopped by the addition of 200 µL of ethanol vortexed for 60 s, and then centrifuged at 20,000 g for 10 min. Analyses of reaction products were performed by C18 column reverse-phase high-performance liquid chromatography (HPLC) with an Agilent 1100 system (Agilent Technologies, CA, USA) at room temperature. Glucosides of phenol or hydroquinone were separated from their aglycones by a linear gradient of 10% to 30% acetonitrile in water (H_2_O) at 1.0 mL/min for 10 min and monitored at 213 nm. For analyzing the glucosides of catechol, a linear gradient of 5% to 15% acetonitrile in water (H_2_O) for 15 min was used as mobile phase. To confirm the glucosides of phenol and hydroquinone, phenyl β-D-glucopyranoside (PGPS) and arbutin (both from Sigma) were analyzed by reverse-phase HPLC. Since the glucosides of catechol were not available, liquid chromatography-mass spectrometry (LC-MS) analyses were performed to determine the products of catechol. 30 µL of the whole reaction mixture was applied to LC-MS. Glucosylated catechol was separated from its aglycone by a linear gradient of 10% to 30% acetonitrile in H_2_O at 1.0 mL/min for 10 min and monitored at 213 nm. The peak corresponding to the product was subjected to mass spectrometry analysis. Negative ion mass spectrometry analyses were performed with electrospray ionization source on 3900 V, 10.0 L/min drying gas flow, 40 psi nebulizer pressure, and 350°C drying gas temperature.

### Determination of Kinetic Parameters for PtUGT72B1 Towards Phenol, Hydroquinone, and Catechol

About 10 µg of crude heterologously expressed PtUGT72B1 protein extracts was used to determine apparent *Km*. The assay mixtures were similar to those described above, except aglycones range from 0.1 mM to 0.7 mM for phenol and catechol, and from 0.4 mM to 1.6 mM for hydroquinone.

### Tests of Yeast and Plant Tolerances

To test yeast tolerance, exponentially growing cells were harvest and transferred in BMMY medium supplemented with 1% methanol to induce expression for 24 h. The cultures were then diluted to A_600_ 0.1 and spotted on solid BMGY medium containing 9.0 mM phenol, 0.9 mM hydroquinone, or 8.2 mM catechol.

*PtUGT72B1* gene was introduced into *A. thaliana* (ecotype Colubia) under the control of the cauliflower mosaic virus 35S promoter for overexpression [22]. Seeds of wild-type (WT) *A. thaliana* and three T_2_ homozygous PtUGT72B1 transgenic (PT) lines (PT-2, PT-5, and PT-6) that showed high tolerance towards TCP in our previous study were geminated on MS medium containing 0 µL or 10 µL ethanol (as control), 0.37 mM or 0.48 mM phenol, 0.36 mM or 0.45 mM hydroquinone, and 0.18 mM or 0.27 mM catechol for 2 wk. Seeds of WT *A. thaliana* were also grown on MS plates containing 0.48 mM phenol or PGPS, 0.45 mM hydroquinone or arbutin vertically for 1 wk before documentation of the phenotype. Each treatment was performed thrice. The root lengths of all seedlings from each treatment were measured.

### Enzyme Preparation from Transgenic*A. thaliana* and Activity Assays

Leaves from 4-week-old PT plants (PT-2 and PT-5) and WT plants (0.05 g fresh weight) were ground to a fine powder in a 1.5 mL Eppendorf centrifuge tube with liquid nitrogen. The powder was immediately suspended in 500 µL of pre-cooled 50 mM PBS (pH 7.4) and incubated on ice for 30 min. Cell debris was then removed by centrifugation at 20,000g for 20 min at 4°C. The supernatant was used as the crude enzyme extract. Total protein concentration was determined by the Bradford method [30].

The assay mixtures (200 µL) contained 50 mM PBS (pH 7.4), 1 mM DTT, 0.1 mM EDTA, 2 mM UDP-glucose, 50 µg of crude enzyme, and 0.2 mM aglycone (4 µL of each 10 mM stock solution in ethanol). After incubation at 30°C for 15 h, the reaction was stopped by addition of 200 µL of ethanol, vortexed for 60 s, and centrifuged at 20,000 *g* for 10 min. The supernatant was analyzed by reverse-phase HPLC. Glucosides of phenol and catechol were separated from their aglycones by a linear gradient of 10% to 30% acetonitrile in H_2_O at 1.0 mL/min over 10 min and monitored at 213 nm. For hydroquinone, a linear gradient of 5% to 10% acetonitrile in water was used as the mobile phase.

### Feeding Studies with Phenolic Compounds and PGPS

Twenty 1-week-old WT and PT seedlings were separately transferred to 50 mL flasks containing 20 mL of liquid MS medium with 3% (weight/volume) sucrose at 24°C with a 16 h/8 h light cycle on an orbital shaker (60 rpm). After 7 d, the liquid media were refreshed with 20 mL of new MS liquid medium containing 1.06 mM phenol, 0.45 mM hydroquinone, or 0.18 mM catechol (dissolved in ethanol); 20 µL of ethanol was used as a control. The concentrations of phenol and its glucosides in the growth medium were determined every 24 h by reverse-phase HPLC as described above. For hydroquinone or catechol, determination was done every 3 h.

To determine the glucosides content of phenol, hydroquinone, and catechol in plants, PT or WT plants incubated with phenol for 24 h or with hydroquinone or catechol for 6 h were obtained, washed thrice with distilled water, and then dried to a constant weight at 65°C. The dried plants were then ground to a fine powder and extracted with 80% ethanol overnight. After centrifugation at 20,000 g for 20 min, the supernatant was dried in vacuum, dissolved in H_2_O, filtered through a 0.45-µm filter (Millipore), and then analyzed by reverse-phase HPLC. Since the catechol-glucoside standard was not available, the relative content of glucosides in plants was presented as the area of the peak obtained during HPLC analysis per gram of dry plants (AU·s/g).

To determine the absorbance efficiency of PGPS and arbutin, 2-week-old WT plants were transferred to new MS liquid medium containing 1.06 mM PGPS or 0.45 mM arbutin (dissolved in sterilized water). Determination of the PGPS and arbutin contents in the media and plants was performed after 1 d and 6 h, respectively.

Isolation and analysis of soluble reducing sugar content in WT and PT plants.

To determine the reducing sugar content in the plants, 1-week-old WT and PT (PT-2 and PT-5) plants were removed from the plates and treated with ethanol (as a control), phenol, hydroquinone, or catechol in the liquid medium for 3 d. The plants were then washed thrice with distilled water and dried with absorbent papers. The plants were ground with distilled water in a mortar, heated for 20 min at 50°C and mixed with 50 mL of H_2_O. After centrifugation at 20,000 g for 20 min, the supernatants were used for reducing sugar content determination. The reducing sugars were estimated as glucose equivalents by the dinitrosalicylic acid (DNS) [31].

## Results

### GT Activity of PtUGT72B1*in vitro*

**Figure 1:**
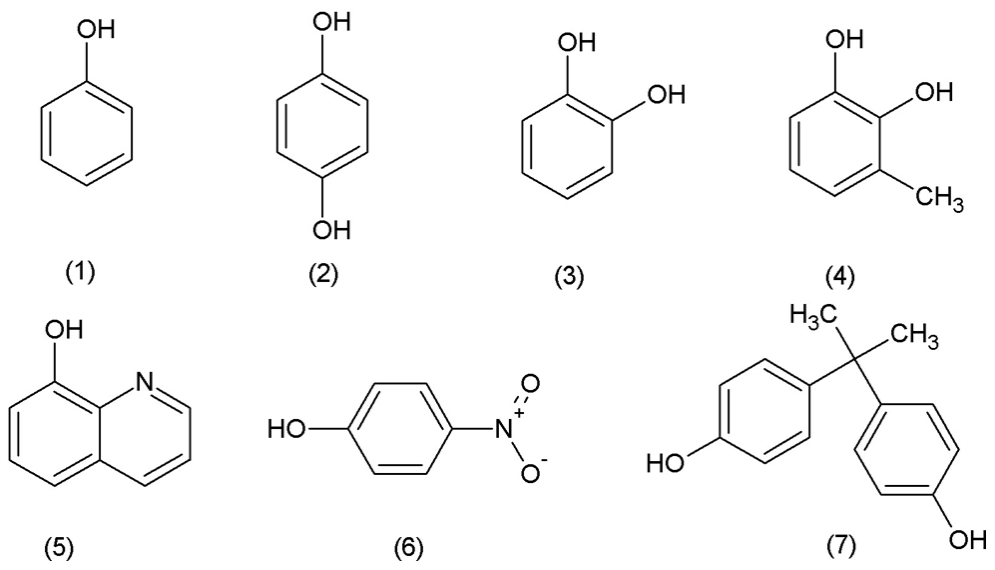
Acceptor substrates used to determine the substrate preferences of PtUGT72B1: (1) phenol, (2) hydroquinone, (3) catechol, (4) 3-methylcatechol, (5) 8-hydroxyquinoline, (6) 4-nitrophenol, and (7) bisphenol A.

**Figure 2:**
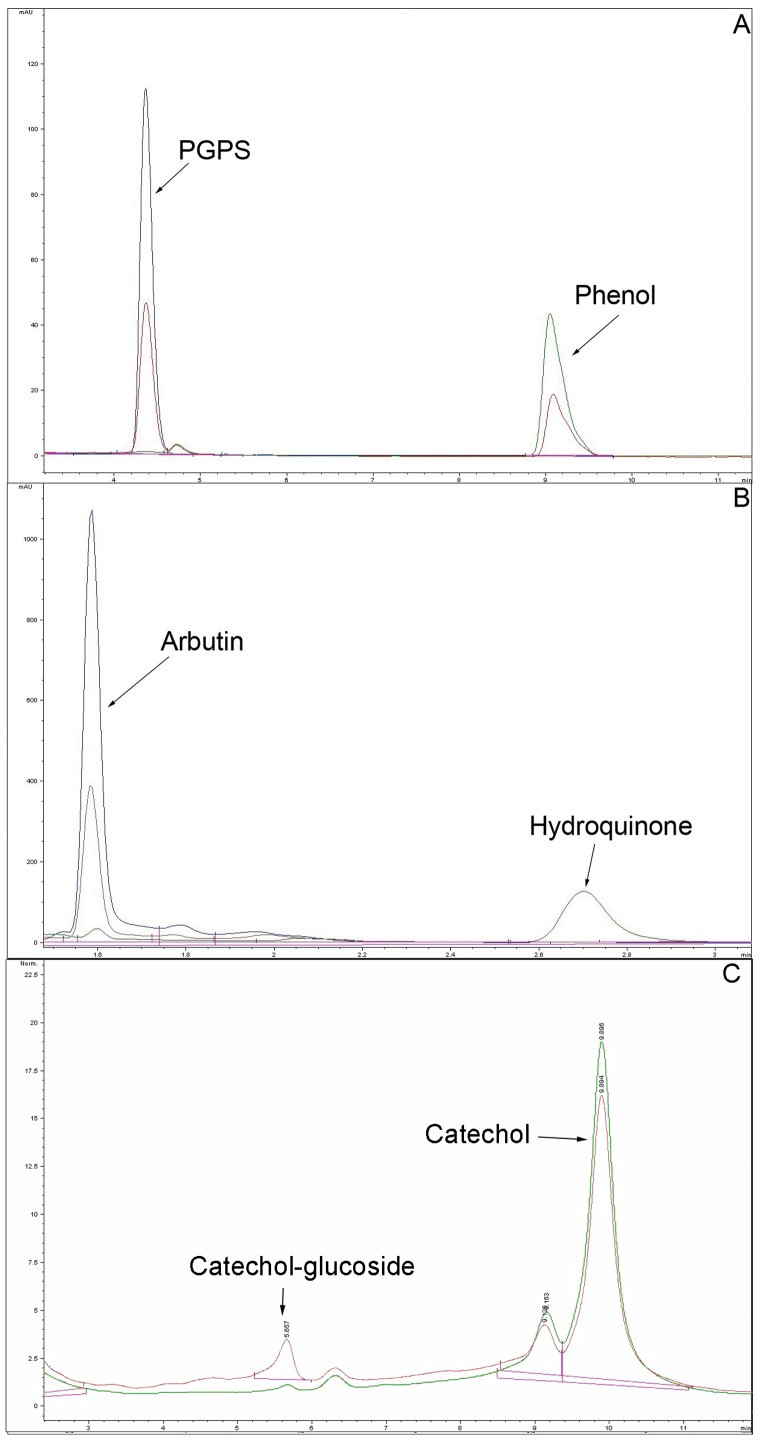
HPLC analyses of PGPS, arbutin, and products from phenols incubated with UDPG and protein extract from*Pichia pastoris*. (A) PGPS authentic standards (blue) and phenol incubated with UDPG and protein extract of *P. pastoris* expressing PtUGT72B1 (red) or containing empty vector (green). (B) Arbutin authentic standards (blue) and hydroquinone incubated with UDPG and protein extract of *P. pastoris* expressing PtUGT72B1 (red) or containing empty vector (green). (C) Catechol incubated with UDPG and protein extract of *P. pastoris* expression PtUGT72B1 (red) or containing empty vector (green).

Recombinant PtUGT72B1 was extracted from *P. pastori*s. The PtUGT72B1 content of the extract reached over 50% of the total protein content after induction for 24 h [22]. The structures of the phenols tested are shown in Fig. 1. After incubation with phenol and UDPG, a new product identical to that of the PGPS authentic standard was observed (Fig. 2A). This product was not present in the control reaction. The content of phenol in the mixture containing PtUGT72B1 appeared to be less than that of the control.

PtUGT72B1 activities against hydroquinone and catechol were also assayed. The new product was observed after incubation of hydroquinone or catechol with UDPG and PtUGT72B1. The product of hydroquinone was identical to that of arbutin (Fig. 2B). A new peak from catechol was also observed (Fig. 2C). To identify the mass corresponding to this peak, LC–MS analysis was performed using the whole mixture. MS signals corresponding to the peak of product were obtained (Figure S1). Ions of m/z 270 (expulsion of 2H^+^) and 271 (expulsion of H^+^) indicate the presence of the catechol-1-*O*-glucoside. Ion series of m/z 141, 307, 308, 314, 334, and 492 were back ground ions. The presence of these unexpected ions could be due to the low concentration of product in the mixture. No new product was observed after incubation of bisphenol A, 8-hydroxyquinoline, 3-methylcatechol, and 4-nitrophenol with UDPG and PtUGT72B1 indicating that PtUGT72B1 has no GT activity towards these phenols (data not shown).

### Resistance to Phenol, Hydroquinone, and Catechol in*P.* *pastoris*

**Figure 3:**
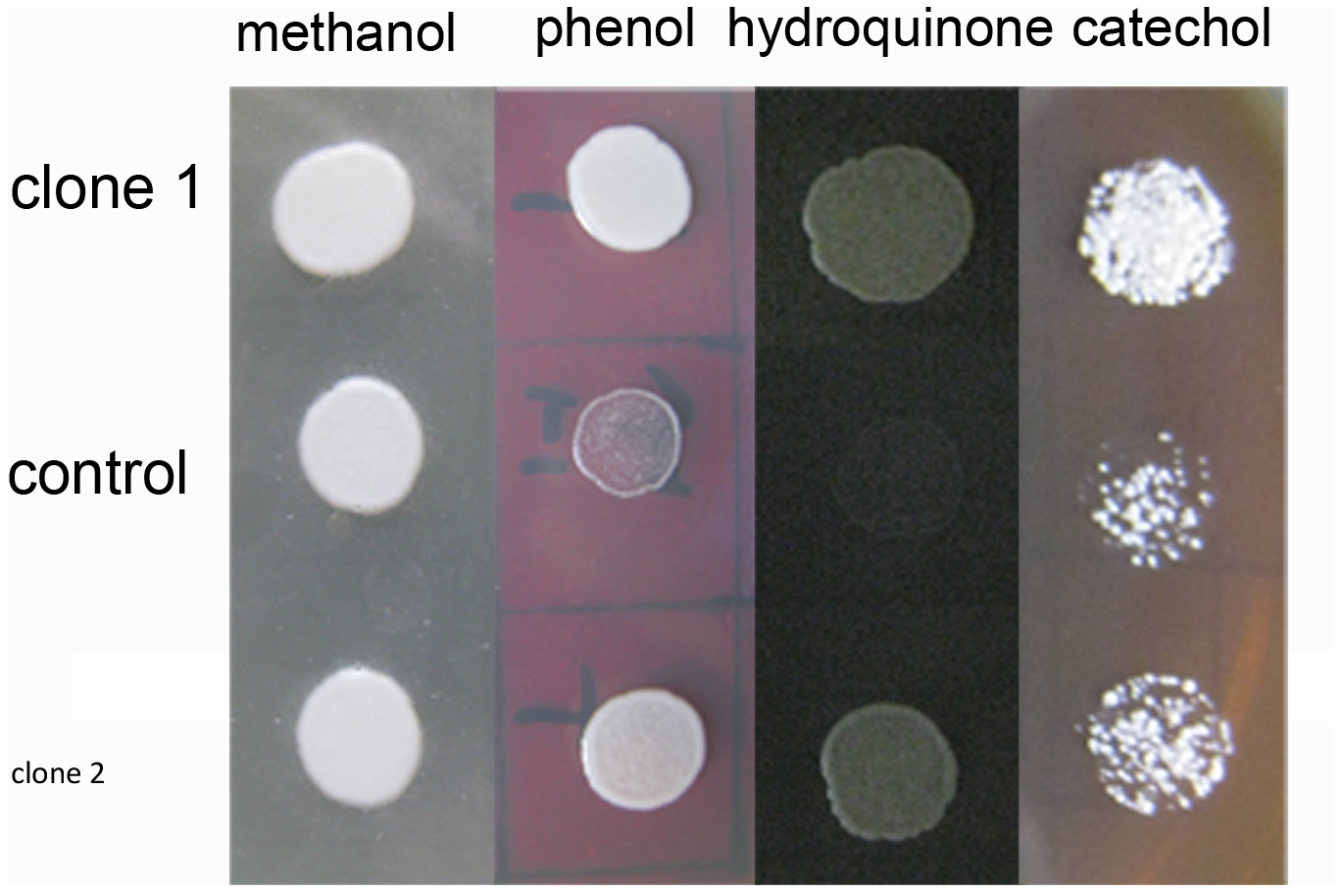
Comparison of the toxicities of phenol, hydroquinone, and catechol to yeast. Two transformant strains of *P. pastoris* that expressed PtUGT72B1 and one transformant strain of *P. pastoris* containing the empty vector (control) were spotted on BMGY plates containing (A) 200 µL methanol, (B) 9 mM phenol, (C) 0.9 mM hydroquinone, and (D) 8.2 mM catechol.

After spotted on BMGY plates containing 9.0 mM phenol, 0.9 mM hydroquinone, or 8.2 mM catechol for 2 d, the growth of yeast cells transformed with empty pPIC9K plasmid was significantly inhibited. Yeast cells transformed with PtUGT72B1 were less inhibited compared with the controls (Fig. 3).

### Kinetic Parameters of the Recombinant GT

The enzyme extract from *P. pastoris* containing empty pPIC9K vector had no GT activity towards hydroquinone and catechol. The kinetic parameters of PtUGT72B1 were studied for acceptor substrates and all data were determined and averaged from three independent assays. The apparent *Km* for PGPS and arbutin formation were 0.286 mM and 0.715 mM respectively. The apparent *Km* for catechol glucosyaltion was 0.161 mM.

The GT activity of the enzyme incubated with 0.2 mM phenols is shown in Table 1. Since the catechol-glucoside standard was not available, the GT activity was presented as the peak area obtained during HPLC analysis per minute per milligram protein (mAU·s/min/mg).

**Table 1:** HPLC analyses of the GT activity of protein extracts from*Arabidopsis* plants and *P. pastoris*.

	Catechol (mAU·s/min/mg)	Phenol (mmol/min/mg)	Hydroquinone (mmol/min/mg)
WT	4.12±0.09	2.67×10^−7^±1.99×10^−8^	4.51×10^−7^±3.38×10^−8^
PT-2	5.51±0.27**	3.67×10^−7^±1.05×10^−8^**	5.52×10^−7^±1.39×10^−8^*
PT-5	5.74±0.10**	4.16×10^−7^±2.20×10^−8^**	6.29×10^−7^±2.38×10^−8^**
*P.pastoris*	716.83±33.17**	3.06×10^−5^±1.41×10^−6^**	4.70×10^−5^±2.15×10^−6^**

The concentration of all phenols in the mixture is 0.2 mM. The estimated GT activity is expressed in mmol/min/mg for PGPS and arbutin and mAU·s/min/mg for catechol-glucoside formation. Data represent mean ± SD (n ±3). Statistical analysis of differences in PT *Arabidopsis* plants and *P. pastoris* with respect to WT *Arabidopsis* plants is performed using Dunnett’s two-tailed *t*-test. Significant difference is denoted with one (P<0.05) or two (P<0.01) asterisks.

### Determination of GT Activity Towards Different Phenols by Crude Enzyme Extraction of PT Plants

To investigate the glucosylating ability of *A. thaliana* to phenol, hydroquinone, and catechol, the crude enzyme extracts containing GT from PT plants and WT *A. thaliana* plants were determined. New product peaks were observed with phenol, hydroquinone, and catechol after incubation with the enzyme extracts of the plants. The glucosylation products from phenol and hydroquinone were identified as PGPS and arbutin, respectively (figures not shown). GT activities are listed in Table 1. These results demonstrate that PT plants can produce higher levels of PGPS, arbutin, and catechol-glucoside than WT plants.

### Features of*PtUGT72B1* Transgenic *A. thaliana* Plants Under Phenol, Hydroquinone, and Catechol

**Figure 4:**
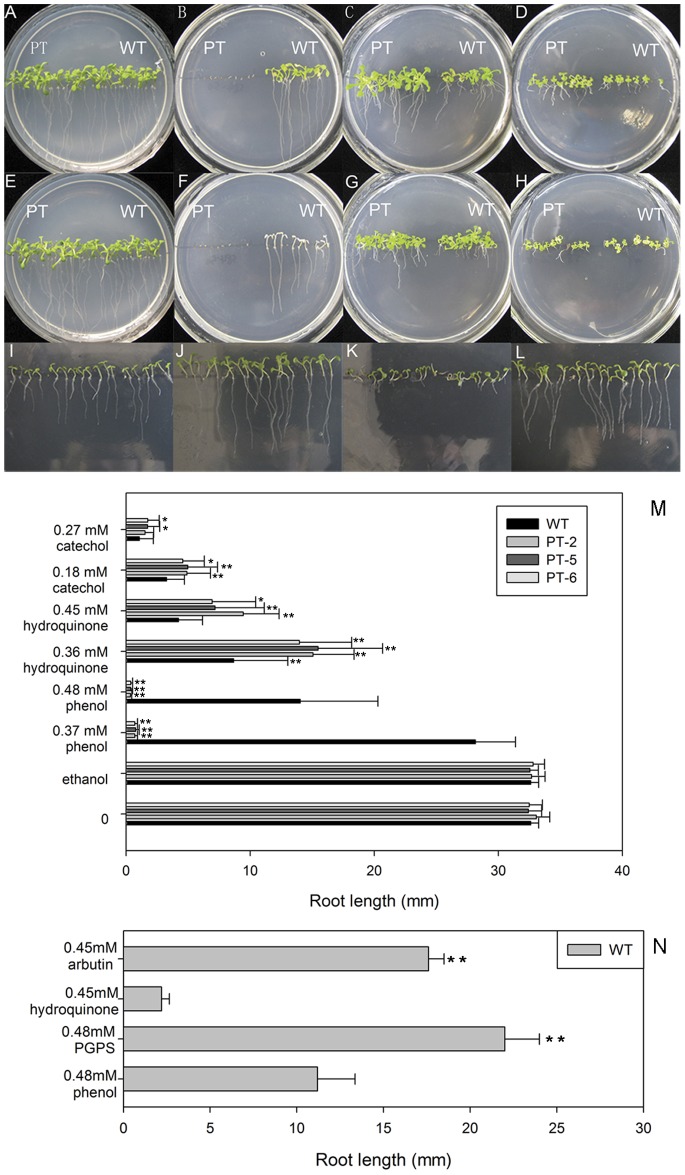
Comparison of the relative toxicities of phenolic compounds and their glucosides to*Arabidopsis.* WT and PT-2 plants germinated and grown vertically for 2 wk on MS agar plates containing (A) 0 µL or (E) 10 µL ethanol, (B) 0.37 mM or (F) 0.48 mM phenol, (C) 0.36 mM or (G) 0.45 mM hydroquinone, and (D) 0.18 mM or (H) 0.27 mM catechol. WT plants germinated and grown vertically for 1 wk on MS agar plates containing (I) 0.48 mM phenol, (J) 0.48 mM PGPS, (K) 0.45 mM hydroquinone, or (L) 0.45 mM arbutin. (M) Root length of 2-week-old WT plants and transgenic plants (PT-2, PT-5, and PT-6) grown on MS agar plates containing different concentrations of various phenols. The data are presented as mean ±SD (n = 30). Statistical analysis of differences in PT lines with respect to WT plants in each treatment is performed using Dunnett’s two-tailed *t*-test. Significant difference is denoted with one (P<0.05) or two (P<0.01) asterisks. (N) Root length of 1-week-old WT plants grown in MS agar plates containing phenol, hydroquinone, and their glucosides respectively. Statistical analysis of differences in WT plants on plates containing phenol or hydroquinone with respect to WT plants on plates containing PGPS or arbutin is performed using Dunnett’s two-tailed *t*-test respectively. Significant difference is denoted with one (P<0.05) or two (P<0.01) asterisks.

PT-2, PT-5, and PT-6 seeds of transgenic *A. thaliana* lines with WT *A. thaliana* plants were grown on MS medium containing different concentrations of phenols for 2 wk. The growth conditions of PT-5 plants are shown in Fig. 4. In the control media without phenols, PT and WT plants appeared equally healthy with similar root lengths and developed leaves (Figs. 4A, 4E, and 4M). The cotyledons of WT plants were green in the medium containing 0.37 mM phenol but began to bleach before true leaves had formed in the medium containing 0.48 mM phenol. Surprisingly, the seeds of PtUGT72B1-expressing lines failed to form cotyledons completely and showed very short roots, indicating that they have lower tolerance to phenol than WT plants (Figs. 4B, 4F, and 4M). All of the PT plants showed significantly higher tolerance in the media containing 0.36 mM hydroquinone, 0.45 mM hydroquinone, or 0.18 mM catechol forming broader leaves, longer petioles, and longer roots than the WT plants (Figs. 4C, 4D, 4G, and 4M). In medium containing 0.27 mM catechol, the average root length of WT plants was significantly lower than that of the PT-5 and PT-6 plants (Figs. 4H and 4M).

The glucosylation products of phenol and hydroquinone are PGPS and arbutin, repsectively. WT seeds were cultured vertically on plates containing phenol, PGPS, hydroquinone, or arbutin for 1 wk. WT plants grown on plates containing 0.48 mM phenol or 0.45 mM hydroquinone for 1 wk showed shorter roots and smaller leaves than plants grown on plates containing 0.48 mM PGPS or 0.45 mM arbutin (Figs. 4I to 4L, and 4N).

### Remediation of Phenol, Hydroquinone, and Catechol by PT Plants

**Figure 5:**
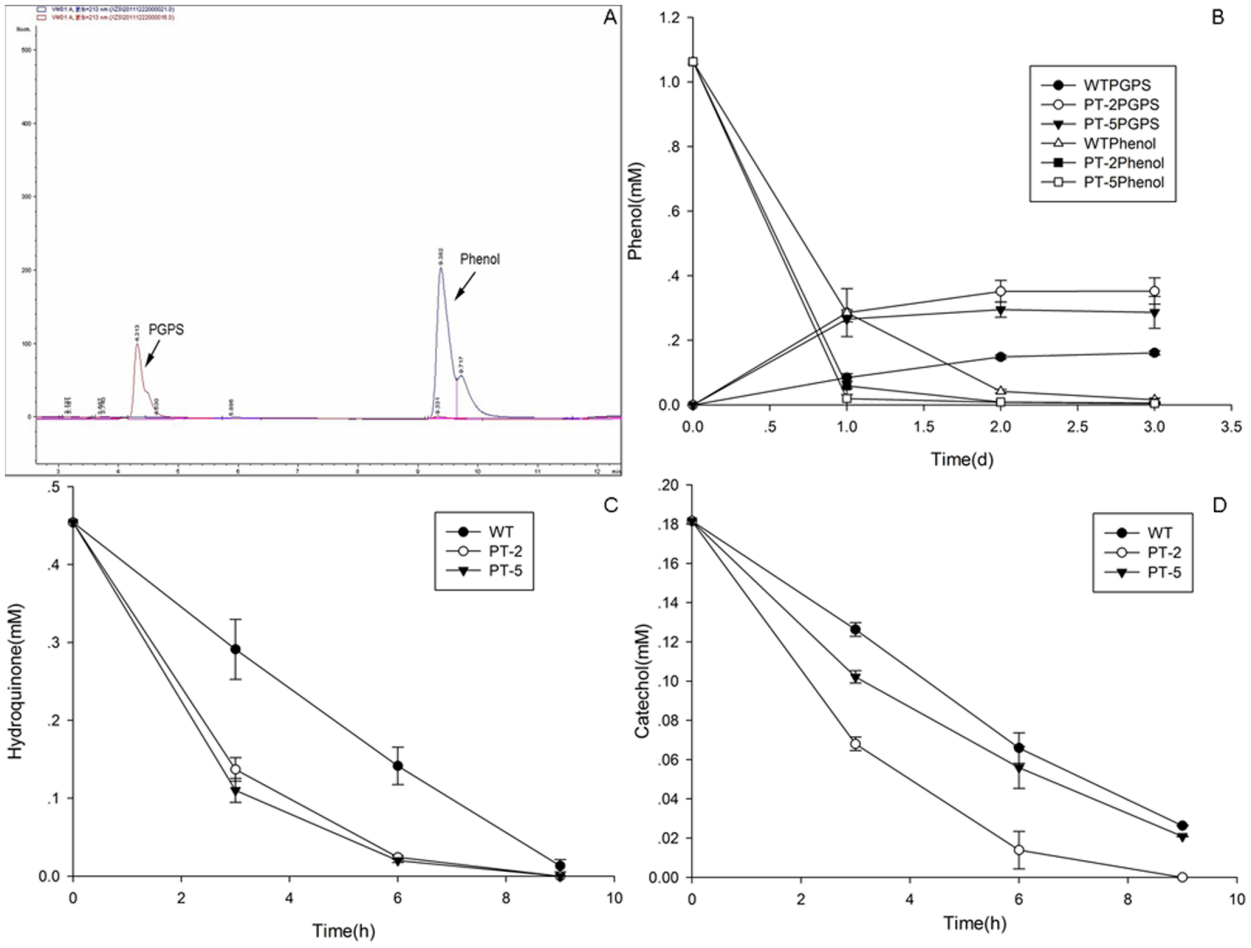
Comparison of the removal efficiencies of*Arabidopsis* plants. (A) A new peak identical to PGPS (red) was observed in the media cultured with PT plants (PT-2) during HPLC analysis. The distributions of (B) phenol and PGPS, (C) hydroquinone, and (D) catechol were determined in the growth medium after WT and PT-2 and PT-5 lines were cultured in MS liquid medium containing 1.06 mM phenol, 0.45 mM hydroquinone, or 0.18 mM catechol at timed intervals. Values are presented as mean ±SD (n = 4).

Since 2-week-old seedlings of WT and PT plants showed higher phenols tolerance than seeds on the plates in our preliminary study (data not shown), the plants were treated with 1.06 mM phenol in liquid MS medium. PT plants treated with phenol grew well but showed slightly lower growth rates compared with WT plants. PGPS was observed in the liquid medium (Fig. 5A). A decreasing amount of phenol and increasing amount of PGPS were found in plants cultured in liquid medium containing phenol. PT plants showed higher removal efficiencies for phenol and excreted more PGPS into the medium than WT plants. After 2 d, phenol was almost undetectable in the medium cultured with PT plants (Fig. 5B).

Compared with phenol, hydroquinone and catechol were degraded faster by plants. PT plants showed higher removal efficiencies for hydroquinone and catechol than WT plants (Figs. 5C and 5D). After 9 h of culture, hydroquinone could not be detected and catechol was mostly degraded.

**Figure 6:**
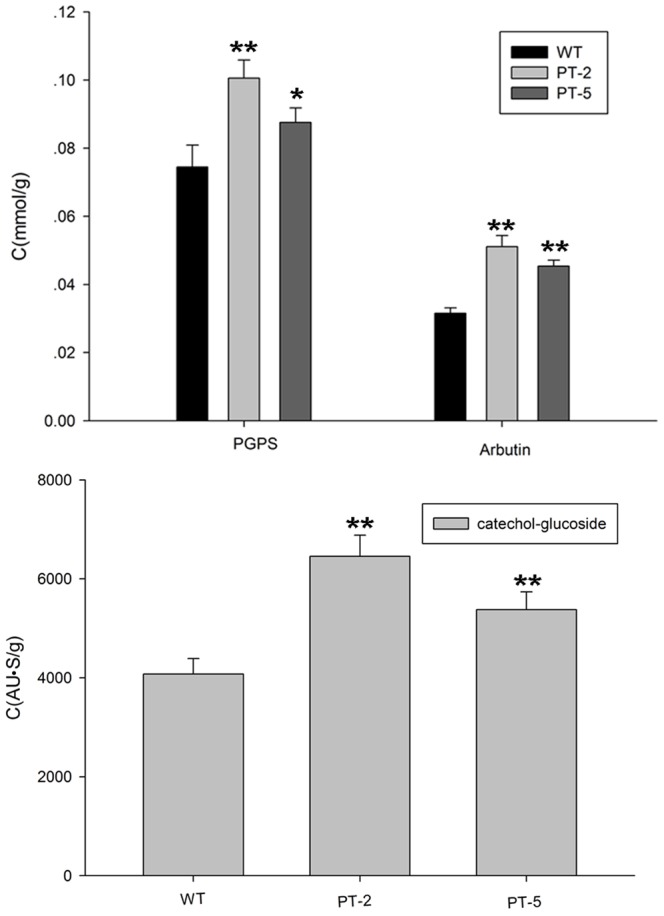
The contents of (A) PGPS and arbutin, and (B) catechol-glucoside in plants after WT and PT-2 and PT-5 lines were cultured in MS liquid medium containing 1.06 mM phenol, 0.45 mM hydroquinone, or 0.18 mM catechol. Values are presented as mean ±SD (n = 4). Statistical analysis of differences in PT lines with respect to WT plants in each treatment is performed using Dunnett’s two-tailed *t*-test. Significant difference is denoted with one (P<0.05) asterisk.

The amounts of PGPS, arbutin, and catechol-glucoside accumulated in either PT or WT plants treated with phenol, hydroquinone, or catechol were analyzed by reverse-phase HPLC. PT plants accumulated more PGPS, arbutin, or catechol-glucoside than WT plants (Fig. 6).

After treatment with 1.01 mM PGPS for 1 d or 0.45 mM arbutin for 6 h, *A. thaliana* plants grew well in the liquid medium. To compare the absorption efficiencies of *A. thaliana* plants towards PGPS and arbutin with that towards phenol and hydroquinone, the contents of PGPS and arbutin remaining in the liquid medium and plants were determined (Table 2). The concentration of PGPS in the medium increased after 1 d while that arbutin remained unchanged after 6 h. The content of PGPS in WT plants cultured with 1.01 mM PGPS, about 1.70×10^−3^ mmol/g dry weight, was much lower than that in plants cultured in the liquid medium containing the same concentration of phenol. The content of arbutin in WT plants cultured with 0.45 mM arbutin, about 0.01 mmol/g dry weight, was much lower than that of plants cultured in the liquid medium containing 0.45 mM hydroquinone.

**Table 2:** Concentrations of PGPS and arbutin left in the media and uptaken by WT*Arabidopsis* plants after culturing 2-week-old WT plants in MS liquid medium containing 1.06 mM PGPS or 0.45 mM arbutin.

	Media (mM)	Plants (mmol/g)
PGPS	1.17±5.14×10^−2^	1.70×10^−3^±9.35×10^−5^
Arbutin	0.45±5.71×10^−3^	0.01±9.37×10^−4^

The concentrations in the media are expressed in mM and the content in the plants are expressed in mmol/g dry weight. The data represent mean ± SD (n = 3).

### Reducing Sugar Content in Plants

**Figure 7:**
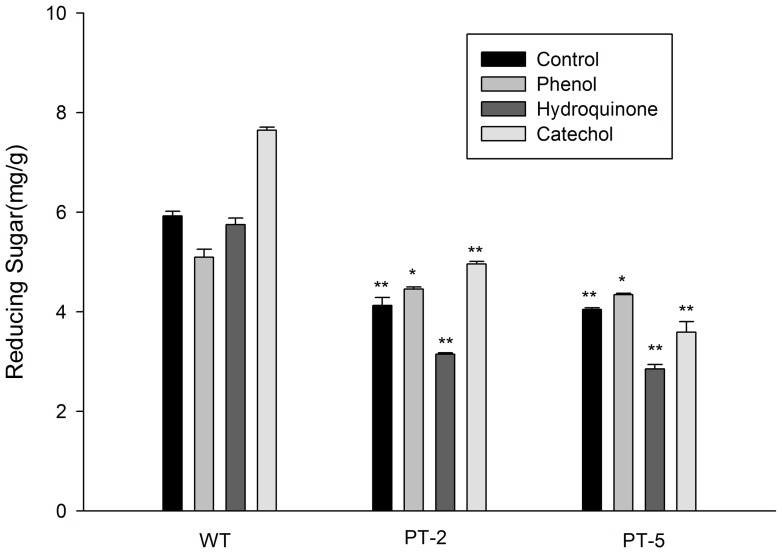
Soluble reducing sugar content in WT and PT*Arabidopsis* plants after the plants were cultured in MS liquid medium containing 1.06 mM phenol, 0.45 mM hydroquinone, or 0.18 mM catechol for 3 d. The data are presented as mean ±SD (n = 3). Statistical analysis of differences between PT lines and WT plants in each treatment is performed using Dunnett’s two-tailed *t*-test. Significant difference is denoted with one (P<0.05) or two (P<0.01) asterisks.

After treatment with phenols for 3 d, the seedlings survived but grew slower than those in media without phenols. PT plants contained less reducing sugar than WT plants in all conditions (Fig. 7).

## Discussion

Plants can glycosylate various low-molecular weight compounds and endogenous and foreign substrates. Among the seven phenols tested, PtUGT72B1 from *P. trichocarpa* was confirmed to catalyze the *O*-glucosylation of phenol, hydroquinone, and catechol when expressed in *A. thaliana* and *P. pastoris*. Phenol, hydroquinone, and catechol [32] have significant toxicities to *A. thaliana*. Plants transform xenobiotics via a three-phase detoxification system: conversion, conjugation, and compartmentalization [33]. Glucosylation of phytotoxic and xenobiotic substances is considered to be part of the phase II detoxification process and enables plants to cope with the enormous diversity of toxic microbial metabolites.

In comparison to WT plants, we found that PT plants could tolerate higher concentrations of hydroquinone and catechol on plates but surprisingly displayed much lower tolerance to phenol. PT plants displayed higher GT activity towards phenol, hydroquinone, and catechol than WT plants both *in vitro* and *in vivo*. These results suggest that the glucoside of phenol, PGPS, is highly intracellular toxic to *Arabidopsis* compared with phenol. However, the phenol in plates was more toxic to *Arabidopsis* plants than PGPS. *Arabidopsis* plants in the liquid medium uptook PGPS much harder than phenol and exported PGPS. Thus the PGPS in the *Arabidopsis* plants cultured with PGPS was maintained at lower concentrations than that produced by glucosylation in the *Arabidop*sis plants cultured with phenol. Germinating PT seeds that failed to survive in the plates containing phenol may have failed to express the adequate exporter to reduce their intracellular PGPS content overproduced by PtUGT72B1. Two-week-old PT seedlings in the liquid medium containing phenol could be less affected because of the expression of this putative exporter. Hydroquinone in plates inhibited the growth of WT plants more effectively than arbutin, indicating that *Arabidopsis* uptakes hydroquinone more effectively than arbutin. Apparent PGPS and arbutin concentrations in the culture medium remained constant probably because the plants poorly absorbed these two glucosides while utilizing the medium for growth.

Sugar conjugation is a major pathway for the inactivation and excretion of both endogenous and exogenous compounds [34]. PT plants can remove phenol, hydroquinone, and catechol from their surrounding media more efficiently than WT plants. The results of this study imply that the *PtUGT72B1* gene can be introduced into plants to enhance their ability to phytodegrade phenolic pollutants. PT plants can glucosylate phenol to PGPS *in vivo* and export it to the surrounding liquid medium more effectively than WT plants. This study is the first to report that *Arabidopsis* plants can glucosylate phenol to PGPS and excrete it. The glucosylation products of hydroquinone and catechol were not detected in the liquid medium but retained in plants. However, whether or not they are transported to the vacuoles for storage or gradually degraded further endogenously is still unknown.

PtUGT72B1 also decreases the reducing sugar content of *Arabidopsis* plants. This finding implies that *Arabidopsis* plants expressing PtUGT72B1 consume more UDPG than plants that do not. UDPG is derived from glucose, which is a kind of the reducing sugar. As WT plants presented higher reducing sugar contents than PT-2 and PT-5 plants in all conditions, an endogenous substrate for PtUGT72B1 must be present in *Arabidopsis* plants.

The activities of PtUGT72B1 towards phenol, hydroquinone, and catechol were confirmed by its heterologous expression in *P. pastoris*. The GT activities of protein extracts from PtUGT72B1-transformed *P. pastoris* towards phenol, hydroquinone, and catechol were significantly higher than those of protein extracts from PT *Arabidopsis* plants suggesting that PtUGT72B1 shows much higher expression levels in *P. pastoris* than that in *Arabidopsis* plants.

Although hydroquinone and catechol carry two hydroxyl groups at different positions around the benzene ring, no other new peaks were detected. The *Km* for arbutin formation of PtUGT72B1 was much higher than that of the hydroquinone-*O*- glucosyltransferase from *Rauvolfia* (*Km*: <1 µM) [35].

Transgenic *P. pastoris* lines showed higher tolerance to phenol, hydroquinone and catechol than the control. No glucosyltransferase activity towards the three phenols was detected in the control. Phenol or hydroquinone supplemented in plates inhibited the growth of *P. pastoris* more effectively than their glucosides. These results prompt us to postulate that PtUGT72B1 can be introduced into *P. pastoris* to detoxify phenols by glucosylation.

In conclusion, PtUGT72B1 can glucosylate phenol, hydroquinone, and catechol and enhance the removal efficiencies of *Arabidopsis* plants towards these phenolic compounds. PtUGT72B1 has direct effects on the toxicities of the phenols to *Arabidopsis* plants and *P. pastoris*.

## Supporting Information

Figure S1 LC-MS analysis of PtUGT72B1 product after incubation with catechol. The whole mixture was used for analysis.
(TIF)
