# Supplementary figures and images for: Selective Detoxification of Phenols by Pichia pastoris and Arabidopsis thaliana Heterologously Expressing the PtUGT72B1 from Populus trichocarpa
Source: PLoS One. 2013 Jun 26;8(6):e66878. doi: 10.1371/journal.pone.0066878 (PMC3694158; doi:10.1371/journal.pone.0066878)

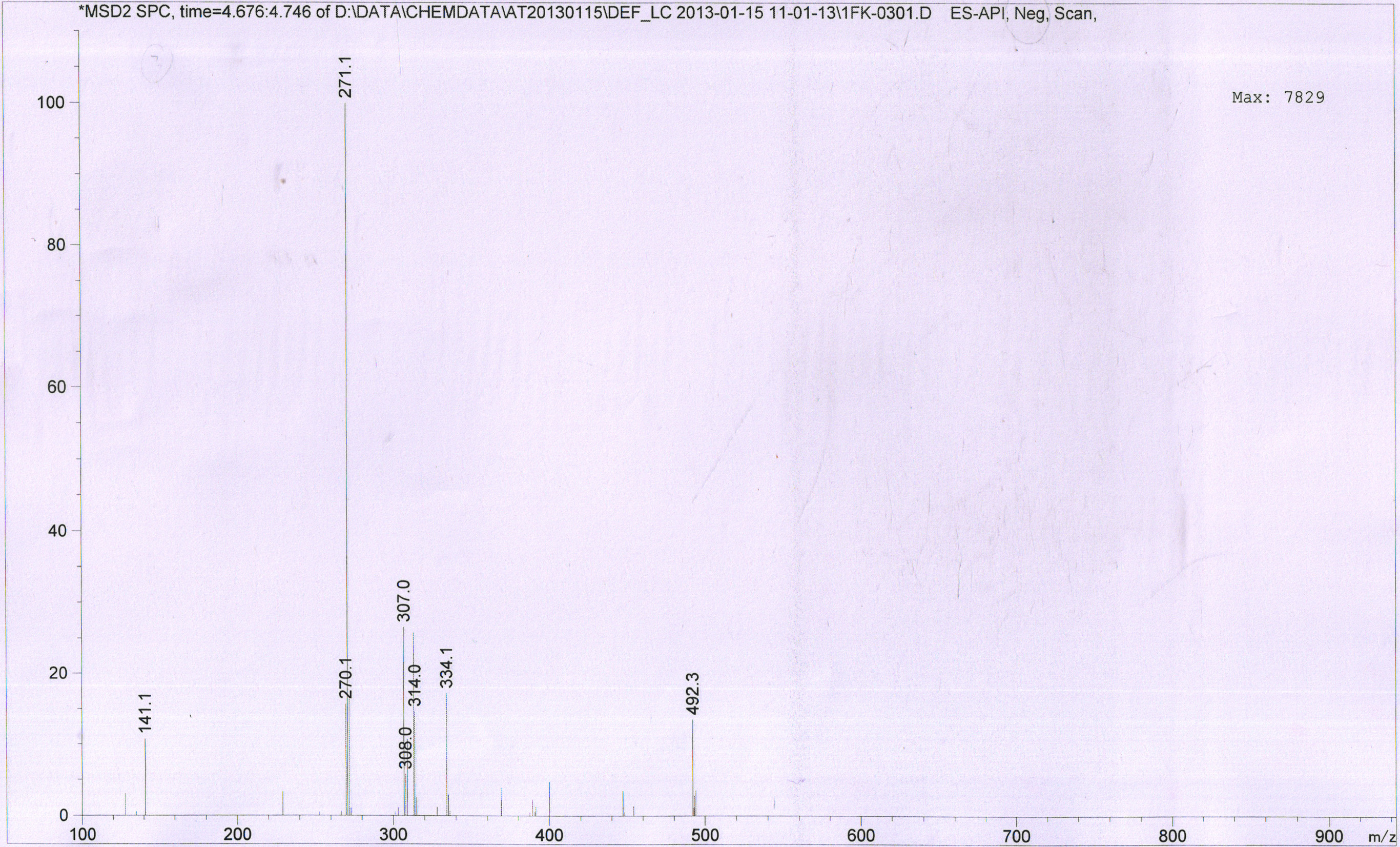

Supplement: Figure S1 — LC-MS analysis of PtUGT72B1 product after incubation with catechol. The whole mixture was used for analysis. (TIF) [file pone.0066878.s001.tif]
